# Theoretical Study of the Structural Stability, Chemical Reactivity, and Protein Interaction for NMP Compounds as Modulators of the Endocannabinoid System

**DOI:** 10.3390/molecules27020414

**Published:** 2022-01-09

**Authors:** Maricruz Rangel-Galván, María Eugenia Castro, Jose Manuel Perez-Aguilar, Norma A. Caballero, Alejandro Rangel-Huerta, Francisco J. Melendez

**Affiliations:** 1 Centro de Investigación, Laboratorio de Química Teórica, Departamento de Fisicoquímica, Facultad de Ciencias Químicas, Benemérita Universidad Autónoma de Puebla, Edif. FCQ10, 22 Sur y San Claudio, Ciudad Universitaria, Col. San Manuel, Puebla C.P. 72570, Mexico; maricruz.rangel@alumno.buap.mx (M.R.-G.); jmanuel.perez@correo.buap.mx (J.M.P.-A.); 2 Centro de Química, Instituto de Ciencias, Benemérita Universidad Autónoma de Puebla, Complejo de Ciencias, ICUAP, Edif. IC8, 22 Sur y San Claudio, Ciudad Universitaria, Col. San Manuel, Puebla C.P. 72570, Mexico; 3 Facultad de Ciencias Biológicas, Benemérita Universidad Autónoma de Puebla, Edif. BIO1, 22 Sur y San Claudio, Ciudad Universitaria, Col. San Manuel, Puebla C.P. 72570, Mexico; norma.caballero@correo.buap.mx; 4 Facultad de Ciencias de la Computación, Benemérita Universidad Autónoma de Puebla, Edif. CCO2, 22 Sur y San Claudio, Ciudad Universitaria, Col. San Manuel, Puebla C.P. 72570, Mexico; arangelfcc@gmail.com

**Keywords:** NMP compounds, DFT calculations, structure stability, chemical reactivity, T-type calcium channel blockers, molecular docking

## Abstract

The cannabinoid receptors (CB1/CB2) and the T-type calcium channels are involved in disorders associated with both physiological pain and depressive behaviors. Valuable pharmacological species carbazole derivatives such as the NMP-4, NMP-7, and NMP-181 (Neuro Molecular Production) regulate both biological entities. In this work, DFT calculations were performed to characterize theoretically their structural and chemical reactivity properties using the BP86/cc-pVTZ level of theory. The molecular orbital contributions and the chemical reactivity analysis reveal that a major participation of the carbazole group is in the donor-acceptor interactions of the NMP compounds. The DFT analysis on the NMP compounds provides insights into the relevant functional groups involved during the ligand-receptor interactions. Molecular docking analysis is used to reveal possible sites of interaction of the NMP compounds with the Ca_v_3.2 calcium channel. The interaction energy values and reported experimental evidence indicate that the site denominated as “Pore-blocking”, which is formed mainly by hydrophobic residues and the T586 residue, is a probable binding site for the NMP compounds.

## 1. Introduction

The endocannabinoid system has been considered the first therapeutic target for physiological pain treatments [1,2], and additionally is involved in mood regulation, major depressive disorders, and appearance of suicidal behaviors [3]. In fact, two of the most relevant proteins involved in disorders associated with physiological pain and depressive behaviors are the cannabinoid receptors (CB1/CB2) and the T-type calcium channels. Particularly, the T-type calcium channels are divided into three isoforms, Ca_v_3.1, Ca_v_3.2, and Ca_v_3.3 channels, and from these, it is the Ca_v_3.2 channel that regulates the neural excitability in primary afferent pain fibers in the presence of nociceptive and neuropathic pain. Furthermore, the Ca_v_3.2 isoform contributes to dorsal root neurotransmission, which is involved in disorders associated to pain and depression [4,5,6]. Recently, it was reported that a significant increase in bursting activity in the lateral habenula neurons, involving the T-type calcium channels, is associated with the appearance of depression [7]. Due to their potential pharmacological role, ligands able to regulate the proteins associated with the endocannabinoid system have been explored. Carbazole derivatives, which were isolated from *Murraya* genus plants [8], present broad anti-inflammatory, antiepileptic, and analgesic biological activities [9]. Several of these compounds, such as Neuro Molecular Production carbazole derivatives, NMP-4, NMP-7, and NMP-181, display a dual effect, acting not only in the CB1/CB2 cannabinoid receptors, but also in the T-type calcium channels. For instance, the NMP compounds can block the Ca_v_3.2 isoform in a micromolar concentration range. In particular, the NMP-181 inhibits Ca_v_3.2 channel with an IC_50_ of 4.5 µM [10], while NMP-4 and NMP-7 block this channel with an IC_50_ of 2.47 µM and 1.84 µM, respectively [11,12]. In this work, the objective is to characterize the structure and chemical reactivity descriptors of NMP compounds, as well as molecular docking analysis to suggest potential interaction sites with the Ca_v_3.2 calcium channel. The structural stability and chemical reactivity properties of the semi-rigid NMP compounds are calculated by using Density Functional Theory (DFT). It was established that BP86/cc-pVTZ is the best-suited level of theory, since it accurately reproduces experimental ^1^H and ^13^C NMR chemical shifts [10,11]. NMP compounds were obtained as unique minima for NMP-4, NMP-7, and NMP-181 on the potential energy surface (PES). Electronic properties such as the frontier molecular orbitals (FMO) distributions and molecular electrostatic potential (MEP) maps were then analyzed. Natural bond orbital (NBO) analysis was used to determine the stability of the compounds. Reactivity analysis was determined by global and local reactivity descriptors, which are used to identify the possible regions of the interaction of these compounds with the receptor proteins. Finally, molecular docking calculations were made to establish the possible binding sites of NMP compounds in the Ca_v_3.2 channel.

## 2. Results and Discussion

### 2.1. Molecular Structure of NMP Compounds

**Figure 1:**
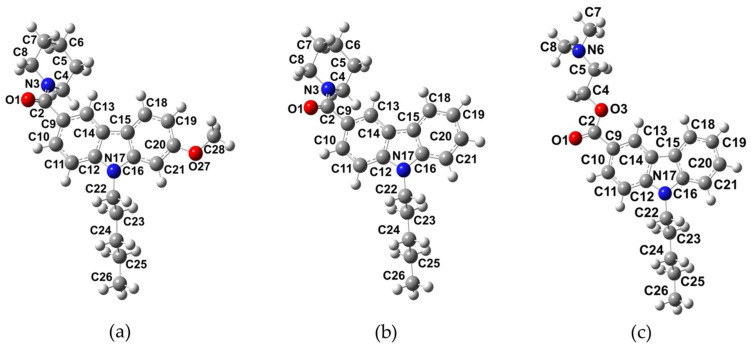
Optimized structures of NMP compounds: (**a**) NMP-4, (**b**) NMP-7, and (**c**) NMP-181 obtained at the BP86/cc-pVTZ level of theory using chloroform as solvent.

NMP compounds were obtained as the lowest energy structures on the PES after conformational analysis, as shown in Figure 1. The NMP compounds share structural similarities including the carbazole group, the pentyl group, and the carbonyl group. In the NMP-4 and NMP-7 compounds, a piperidine ring is attached to the carbonyl group (C2=O1), while for NMP-181 the ester and terminal amine groups are attached to the carbonyl group (C2=O1). The NMP-4 compound bears a methoxy group attached to the carbazole group (at C20 atom), as shown in Figure 1. Results in chloroform as the ligand solvent are especially important since a non-polar environment is desirable to better simulate the environment found in the binding pockets of the specific target receptor proteins of these compounds (CB1/CB2 receptors and T-type calcium channels).

Table 1 shows selected bond lengths, valence angles, and dihedral angles of the NMP compounds, as obtained at the B98/cc-pVTZ level of theory. Atom labels correspond to those described in Figure 1. The bond lengths and the valence angles do not present any significant changes in the three compounds. The most significant differences are found on the dihedral angles involved in the piperidine group attached to the carbazole group in NMP-4 and NMP-7, and the dihedral angles in the ester and amine groups in NMP-181. For NMP-4 and NMP-7, the values for the dihedral angle O1−C2−N3−C4 are −159.8° and −161.14°, for the C5−C4−N3−C2 dihedral angle are −138.17° and −135.70°, and for the C9−C2−N3−C4 dihedral angle are 22.16° and 20.78°, respectively; observing that in both compounds the position of the piperidine moiety is preserved. For NMP-181 the dihedral angles O1−C2−O3−C4, C5−C4−O3−C2, and C9−C2−O3−C4 indicate the position of the ester and amine group with values of 0.0°, 174.1°, and −179.8°.

In general, these results show that the semi-rigid structure of the NMP compounds is kept in similar conformation for the common functional groups: carbazole, pentyl, and the carbonyl group. The changes observed are a consequence of the piperidine in NMP-4 and NMP-7, and the ester and amine groups in NMP-181.

### 2.2. NMR and IR Calculations

#### 2.2.1. ^1^H and ^13^C NMR

The ^1^H and ^13^C NMR chemical shifts (δ) for the NMP compounds were calculated to compare with those experimental values reported in the literature [10,11]. The values of δ obtained with the level of theory BP86/cc-pVTZ are in good agreement with the experimental data. Tables S1 and S2 show the main ^1^H and ^13^C NMR δ values, respectively, obtained for the three NMP compounds.

For ^1^H NMR, similar δ for H_ring_ of the carbazole group are observed for NMP-7 and NMP-181 with values of 7.51–9.00 ppm, while for NMP-4, the values decrease to 6.98–8.18 ppm due to the presence of the methoxy group. δ for H_R-CH2-N_, H_CH2_, and H_CH3_ of the pentyl group are 4.24–4.43, 1.40–1.87, and 0.93–1.22 ppm, respectively, for the three NMP compounds. The methoxy group in NMP-4 shows a δ in H_CH3O_ 3.89–4.11 ppm and the amine and ester groups in NMP-181 for H_CH3-N_ and H_CH2-O_ have δ values of 1.73–2.83 and 4.15–4.71 ppm, respectively; see Table S1.

**Figure 2:**
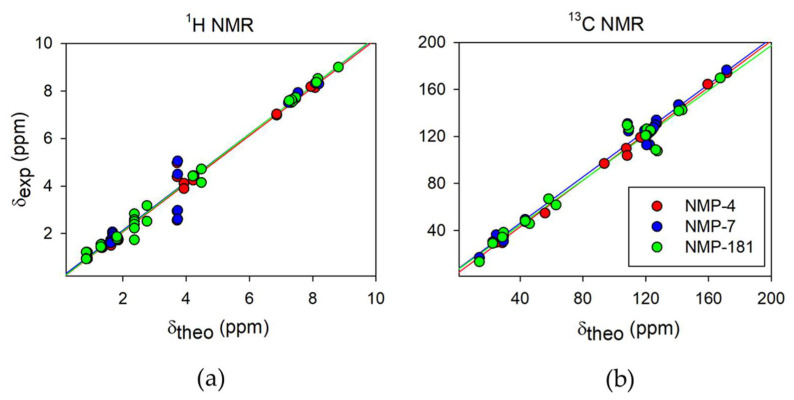
Correlation graphs of calculated δ values (in ppm) with the experimental δ values. In: (**a**) ^1^H and (**b**) ^13^C NMR for the NMP compounds obtained at BP86/cc-pVTZ level of theory in chloroform.

For ^13^C NMR, δ values for C_ring_ at the carbazole group are 121.0–131.1 ppm. In the pentyl group, δ values for C_CH2-N_, C_CH2-C_, C_CH3-C_ are obtained at 47.4–62.8, 29.4–35.3, and 16.5–16.6 ppm, respectively. δ value for C_C-OCH3_ at the methoxy group in NMP-4 is 164.4 ppm. δ values for C_CH3-N_ and C_CH2-O_ of the amine and ester groups in NMP-181 are 47.0–51.7 and 69.0 ppm; see Table S2. All the calculated δ values agree with the experimental data reported. The major difference in the calculated data for ^1^H and ^13^C NMR are 1.49 and 14.73 ppm, respectively, regarding the experimental data in chloroform [10,11]. Some of the major differences of calculated chemical displacements with respect to the experimental data [10,11] were those H or C that were close to electronegative atoms (N or O) and, therefore, were more deshielded and susceptible to interaction with the solvent. Several factors could be involved in addition to the electronegativity, such as the molecular geometry, the inductive effect, electron delocalization, etc. [13]. For example, in NMP-4 and NMP-7 compounds, the protons bonded at C8 near the carbonyl oxygen had a difference of 1.32 ppm, while in NMP-181 the proton bonded in C10 and C8 had a major difference of 0.63 ppm. Further, the C20, C9, C4 had a difference of 4.97, 4.01, and 10.88 ppm for NMP-4, NMP-7, and NMP-181, respectively (see Figure 1 for labelling). Figure 2 presents the correlation graphs of calculated δ values obtained at BP86/cc-pVTZ level of theory with the experimental δ values for NMP compounds. Figure 2a shows R^2^ values of 0.974–0.992 for ^1^H and Figure 2b shows R^2^ values of 0.973–0.997 for ^13^C NMR. In general, the results are satisfactorily obtained for δ ^1^H and ^13^C NMR in NMP compounds.

#### 2.2.2. IR Characterization

The IR characterization was carried out at the same level of theory, BP86/cc-pVTZ. Table S3 collects the most representative frequencies, intensities, scaled frequencies with 1.014 factor, and the Potential Energy Distribution (PED ≥ 10%). Table S3 shows the characteristic bands for NMP compounds. The most intense vibration is assigned to the C=O stretching located at 1623.2, 1619.5, and 1692.1 cm^−1^ for NMP-4, NMP-7, and NMP-181, respectively. This last value is modified by the ester group in NMP-181 causing that the absorption band increases at a higher frequency. The reported value for C=O stretching of the amide group is in the range 1680–1630 cm^−1^ [14] and of the ester group in 1730–1715 cm^−1^ [15]. The asymmetric and symmetric C-H stretching of the pentyl group is in 3054.3 and 2980.5 cm^−1^, 3055.7 and 2980.9 cm^−1^, and 3055.6 and 2981 cm^−1^, for NMP-4, NMP-7, and NMP-181, respectively. These values are similar to 2926 and 2853 cm^−1^, reported for asymmetric and symmetric stretching of a methylene group, respectively [13]. The symmetric C-H stretching for the methyl group is located at 2992 cm^−1^ for NMP-7 according to the 2872 cm^−1^ reported value for this group [13]. The asymmetric and symmetric C-H stretching for the piperidine group are 3039.3 and 2991.0 cm^−1^ for NMP-4, and 3039.3 and 2989.0 cm^−1^ for NMP-7. The reported value in literature for asymmetric C-H stretching is 3000–2800 cm^−1^ and for the symmetric C-H stretching is 2870–2850 cm^−1^ [15,16,17].

**Figure 3:**
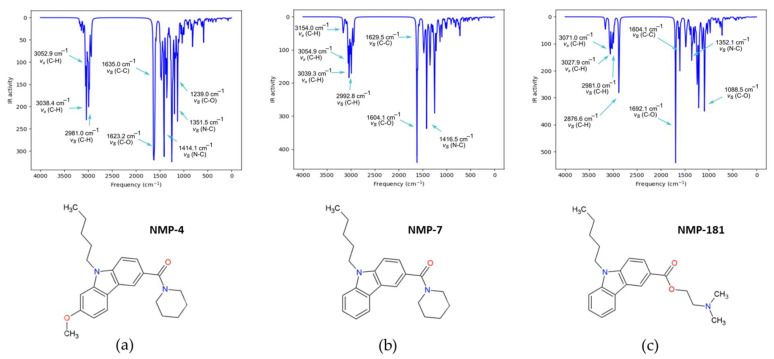
Theoretical IR spectra of NMP compounds: (**a**) NMP-4, (**b**) NMP-7, and (**c**) NMP-181 at BP86/cc-pVTZ level of theory in chloroform.

Additionally, the N-C stretching in the amide group is 1416.5 cm^−1^ and for the pyrrole nitrogen in carbazole group is 1349.9 cm^−1^ for NMP-7. The reported value for these groups is 1400 cm^−1^ and 1342–1266 cm^−1^, respectively [13]. The C=C stretching in the carbazole group is in 1635.0, 1629.5, and 1629.4 cm^−1^ for NMP-4, NMP-7, and NMP-181, respectively. These values are according to the value of 1625 cm^−1^ for the aromatic ring. In NMP-4 the band increases at a higher frequency for the presence of the methoxy group [15]. The C-H stretching in the carbazole group is 3162.3 and 3163.6 cm^−1^ in NMP-7 and NMP-181, respectively. The reported value for alkene C-H stretching is usually observed above 3000 cm^−1^, in the range of 3050–3000 cm^−1^ [15]. For NMP-4 the presence of methoxy group in the absorption region at 3106.0 and 2976.5 cm^−1^ for asymmetric and symmetric stretching of CH_3_ group, respectively, is closed to the reported region at 2830–2815 cm^−1^ [15]. The C-O-C stretching is in 1239.0 cm^−1^, the reported region for ether group is 1300–1000 cm^−1^ [16,17,18]. Furthermore, the 585 cm^−1^ vibration of the O-C-C bending is according to the region 580–505 cm^−1^ for aromatic compounds with methoxy groups [15]. For the NMP-181 the asymmetric and symmetric C-H stretching of amine are at 3079.3 cm^−1^ and 2876.6 cm^−1^, respectively, corresponding to the range of the -N(CH_3_)_2_ group in 2820 and 2770 cm^−1^ [15]. Further, the amine C-N stretching vibration of 1263.7 cm^−1^ is close to the 1270 cm^−1^ reported value for tertiary dimethyl amine [15]. The ester C-O-C stretching vibration appears in 1207.1 and 1088.5 cm^−1^ corresponding to the known range at 1210–1173 cm^−1^ [15,18]. Figure 3 shows the theoretical IR spectra of the NMP compounds with the main characteristic bands assigned.

### 2.3. Electronic Properties

#### 2.3.1. Frontier Molecular Orbitals (FMO)

**Figure 4:**
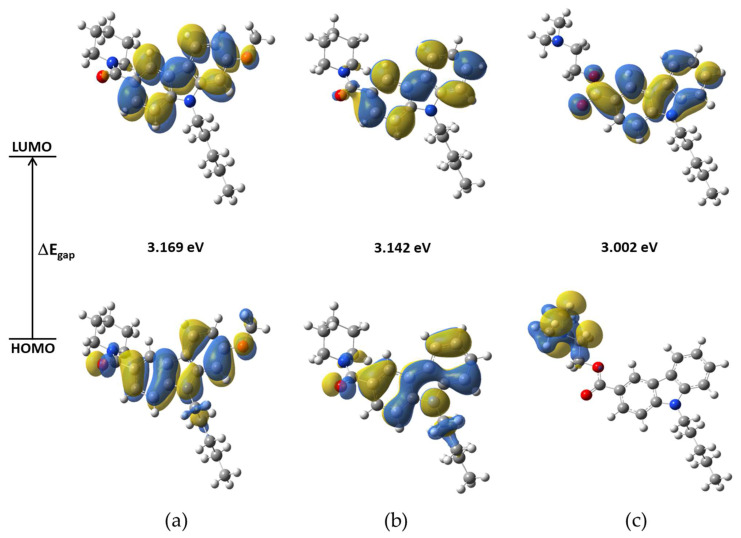
Frontier molecular orbitals (HOMO and LUMO) and ΔE_gap_ values. In: (**a**) NMP-4, (**b**) NMP-7, and (**c**) NMP-181 compounds at BP86/cc-pVTZ level of theory in chloroform.

The FMO are relevant for predicting the relative reactivity based on the electronic structure properties of a molecular system. The chemical properties of a molecule are controlled by the valence orbitals. In this way, nucleophilic attacks are controlled by the HOMO orbital and electrophilic attacks are controlled by the LUMO orbital. The HOMO-LUMO gap energy (ΔE_gap_) is considered as a measure of the molecular structure stability [19]. Table S4 contains the HOMO and LUMO energies, E_HOMO_ and E_LUMO_, and gap energies, ΔE_gap_, for NMP compounds. The ΔE_gap_ is ~3.0 eV at BP86/cc-pVTZ level of theory. NMP-4 has higher gap energy (3.17 eV) than the other NMP compounds; see Figure 4.

Table S5 shows the percentage of the contributions of the functional groups to the molecular orbitals from LUMO+3 to HOMO−3. The carbazole group on the NMP-4, NMP-7, and NMP-181 compounds contributes almost entirely (95%, 97%, and 76%) to the LUMO; the difference observed of 2% for NMP-4 and 21% for NMP-181 with respect to NMP-7 is due to the attached methoxy group to the NMP-4 and the ester group to the NMP-181 compounds. The carbazole group greatly contributes to the HOMO orbital (80% and 86%) for NMP-4 and NMP-7 compounds, respectively, while for NMP-181 the amine group has a major percentage (97%) contribution. The carbonyl group contributes to the HOMO in 8 and 7%, for NMP-4 and NMP-7, respectively, while only participating with 1% in the LUMO in both compounds. In the NMP-181, the ester group participates with 23% in LUMO and only 2% in HOMO. The pentyl group has small contributions on the analyzed orbitals, see Table S5.

Figure 4 shows the isosurfaces of the FMO using an isovalue of 0.02 a.u. It was observed that a great contribution for LUMO is from C atoms of carbazole group and a minor contribution from O atoms in the carbonyl group of NMP-4 and NMP-7 and in the ester group for NMP-181. HOMO major contributions of C and N atoms of carbazole group and minor contributions of C22 and C23 of pentyl group are observed for NMP-4 and NMP-7, while for NMP-181 the HOMO have major contributions of atoms of amine of the piperidine group.

The contribution of the LUMO orbital is located at >75% on the NMP carbazole group, it can be speculated that this molecular region possesses the major participation during the ligand-receptor interaction for both CB1/CB2 receptors and T-type calcium channel. The energies of the FMO can be related to the experimental measurements of compounds determining their biological activity. For example, for ethosuximide, another T-type calcium channel blocker, its gap energies obtained at the HF/6-311+G(d,p) level of theory were correlated with the anticonvulsive activity determined by the logED50 parameter (effective dose 50%) obtaining R^2^ = 0.97 [20]. Here, it is obtained the correlation between ΔE_gap_ energies and the logIC_50_ (maximum inhibitory concentration to the 50%) values of the T-type calcium channel blocker activity reported for the NMP-4, NMP-7, NMP-181 [10,11,12], NMP-144 [11], and *N*-tert-butyl-2-[4-(9-pentyl-9H-carbazole-3-carbo nyl)piperazin-1-yl] acetamide (Compound 10 in reference [21]) blockers. Figure S1 shows the correlation result obtaining ~R^2^ = 0.90. The ΔE_gap_ energy with respect to logIC_50_ follows the trend: NMP-144 (2.971) < NMP-181 (3.002) < Compound 10 (3.031) < NMP-7 (3.142) < NMP-4 (3.169), which correspond to the IC_50_ values: 5.59 µM > 4.60 µM > 3.68 µM > 1.84 µM y 2.47µM, respectively.

#### 2.3.2. Molecular Electrostatic Potential (MEP)

**Figure 5:**
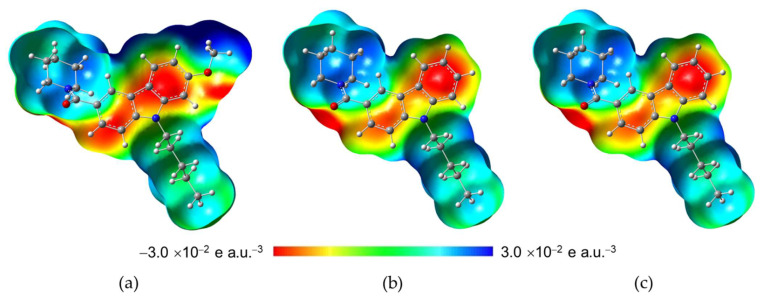
Molecular electrostatic potential maps. In: (**a**) NMP-4, (**b**) NMP-7, and (**c**) NMP-181 compounds at BP86/cc-pVTZ level of theory in chloroform.

The Molecular Electrostatic Potential (MEP) is useful for determining different nucleophilic and electrophilic regions in molecular systems. Figure 5 shows the MEP maps in a range between −3.0 × 10^−2^ and 3.0 × 10^−2^ e a.u.^−3^ for the NMP compounds obtained at BP86/cc-pVTZ level of theory using an isovalue of 0.004 a.u. The major electronic density charge (red region) is located around the O1 in the carbonyl group spread toward the carbazole group in the three NMP compounds; additionally N6 of amine in NMP-181 concentrates major electronic density charge; see Figure 1 for labeling. The deficient electronic density charge (blue region) is on the region where the pentyl group is bonded to N17 of the carbazole group in the three NMP compounds. Furthermore, in the NMP-4 structure deficient electronic density charge region it is observed around H atoms on the methoxy group. In addition, the major electronic density region in the NMP-4 compound extends longitudinally almost 10.14 Å reaching the oxygen in the methoxy group. In the case of the NMP-7, this region measures 8.77 Å in the absence of the methoxy group, and 8.94 Å for NMP-181, similar to the NMP-7 compound. However, a region is located on the N6 atom of the amine group at 5.05 Å (measured from O1 atom of the carbonyl group to N6 of the amine group). It can be proposed as functional similarity of the ligand-receptor electrostatic recognizing and binding pocket coupling for the NMP compounds with the CB1/CB2 receptors and T-type calcium channel [22].

#### 2.3.3. Natural Bond Orbitals (NBO)

The NBO calculations for the NMP compounds were carried out to define a stability measure through the stabilization energy, E (2) parameter. The interaction between donor and acceptor of electrons is larger when the E (2) value increases. The delocalization of electronic density between Lewis-type NBO orbitals (donor) and non-Lewis NBO orbitals (acceptor) corresponds to one stabilizing donor-acceptor interaction [23]. The NMP-7 compound has three hydrogen bond acceptors (O1, N3, and N17), while NMP-4 (O1, N3, N17, and O27) and NMP-181 (O1, O3, N17, and N6) have four of them. Due to the absence of any hydrogen bond donors, in all the NMP compounds, the molecular structure stability cannot be well established by intramolecular hydrogen bond formation. In this case, the NBO analysis is useful to determine relevant interactions between Lewis-type NBO orbitals (donor) and non-Lewis NBO orbitals (acceptor), and to define electronic density delocalization. Table S6 lists the highest stabilization energies E (2) of donors and acceptors for the NMP compounds. The bond (donor) and anti-bond (acceptor) orbitals, π → π*, are found on the carbazole ring carbons in the range at ~11–24 kcal mol^−1^. The bond (donor) and anti-bond (acceptor) orbitals, π (C9–C13) → π* (O1–C2), have larger stabilization energy on the ester group of NMP-181 than on the amide group of NMP-4 and NMP-7. The electronegative atoms O and N interact, through their lone pair electrons, with the antibonding π orbitals of carbon neighbor, LP→ π*, with stabilization energy at ~16–40 kcal mol^−1^, and with the antibonding σ orbital of NMP-181, LP(N6) → σ* (C8–H53), with the energy of 7 kcal mol^−1^. Finally, the anti-bond/anti-bond interaction, π* → π*, corresponds to ~23–84 kcal mol^−1^. The electronic delocalization is observed on the conjugated bonds of the carbazole ring, and it is extended to the region of the lone pairs (LP) of the *p* orbitals of the electronegative neighbor atoms to carbazole moiety. Specifically, in NMP-4 and NMP-7, the electronic delocalization spreads out the amide group (O1 and N3 atoms) and the carbazole group (N17); additionally, on methoxy group (O27 atom) in NMP-4. In NMP-181, the electronic delocalization is observed on the ester group (O1 and O3) and the carbazole group (N17). In this case, in NMP-181, the tertiary amine (N6) contributes to a lesser extent to the delocalization, then it is observed a hyperconjugation interaction. The results state that the stability of the minima energy structures of the NMP compounds is mainly due to the electronic delocalization effect, where the major E (2) values are localized on electronegative atoms and carbazole moiety.

### 2.4. Reactivity Analysis

#### 2.4.1. Global Reactivity Descriptors

Global reactivity descriptors for the NMP compounds were evaluated: chemical potential (μ), electronegativity (χ), hardness (η), softness (s), and electrophilicity index (ω), according to the conceptual DFT approach [24]. Table 2 summarizes the global reactivity descriptors for NMP compounds. The values of the HOMO and LUMO energies are reported in Table S4. In Table 2 it is observed that global reactivity descriptors values in NMP compounds are similar with no significant changes. The methoxy group in NMP-4 slightly decreases the electronegativity and increases the chemical potential by 3.7%, while amine and ester groups in the NMP-181 have an inverse change on those parameters in 1.57% with respect to NMP-7. The hardness is larger in NMP-4, followed by NMP-7 and NMP-181, with values of 3.17 > 3.14 > 3.00 eV, respectively, then NMP-181 decreases the hardness by 4.6% while the NMP-4 increases it by 0.86%, both with respect to NMP-7. The softness keeps a similar value of 0.3 eV in the three compounds. Finally, the NMP-181 has the largest electrophilic behavior, followed by NMP-7 and NMP-4. The methoxy group in NMP-4 decreases the electrophilicity index, while the amine and ester groups of NMP-181 increase this value by ~8% with respect to NMP-7, which does not contain these functional groups; see Figure 1. The electrophilicity index results to be well correlated to the receptor affinity properties and biological activity [25].

#### 2.4.2. Local Reactivity Descriptors

Table S7 shows the condensed Fukui functions, $f^{+}\left(r\right)$ and $f^{-}\left(r\right)$, the dual descriptor, $f^{\left(2\right)}\left(r\right)$, and the Parr functions, $P^{-}\left(r\right)$ and $P^{+}\left(r\right)$, for the NMP compounds. The Fukui functions values are in the ranges of f^+^ = 0.025−0.072 and f^–^ = 0.011−0.070 for NMP-4, f^+^= 0.031−0.072 and f^−^ = 0.012−0.074 for NMP-7, and f^+^ = 0.014−0.086 and f^−^ = 0.002−0.10 for NMP-181. Values of the Fukui functions for relevant functional groups in NMP compounds are f^+^ = 0.048 in the methoxy group O27 (NMP-4) and f^+^ = 0.086 in the amine group N6 (NMP-181). The maxima values indicate the most probable zone for local electrophilic and nucleophilic attacks. The C21, N17, and N6 are the more prone to nucleophilic attack for NMP-4, NMP-7, and NMP-181, respectively, while the C20 is more prone to electrophilic attack for NMP-7, and C13 for NMP-4, and NMP-181. For Parr functions, it is found that the same sites for nucleophilic and electrophilic attack for all compounds; however, the results indicate that C13 is the most probable site for electrophilic attack.

**Figure 6:**
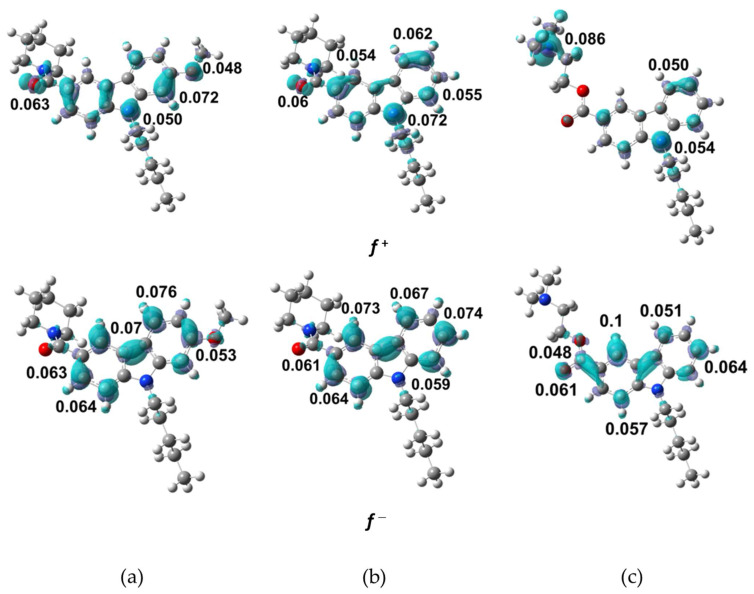
Fukui functions, $f^{+}\left(r\right)$ and $f^{-}\left(r\right).$ In: (**a**) NMP-4, (**b**) NMP-7, and (**c**) NMP-181 compounds at BP86/cc-pVTZ level of theory in chloroform.

Figure 6 presents the isosurfaces for Fukui functions with the major value for f^+^ and f^−^ for NMP compounds, using an isovalue of 0.002 a.u. In this figure, it is observed that f^+^ is located mainly in the carbazol nitrogen N17, in the carbonyl oxygen O1 for NMP-4 and NMP-7, in the methoxy oxygen O27 in NMP-4, and the amide nitrogen N6 in NMP-181. For f^−^ the distribution is located on the carbazol carbons in the NMP compounds and the carbonyl oxygen O1 in NMP-181. In summary, the carbazole group is an important region of electronic density accumulation for the NMP compounds. The calculated distance along this region is 6.6 Å in C9–C20 for NMP-7. It can be proposed functional similarity of the electrostatic recognizing and binding pocket coupling for the NMP compounds with the CB1/CB2 receptors and T-type calcium channel [22].

### 2.5. Molecular Docking Calculations

**Figure 7:**
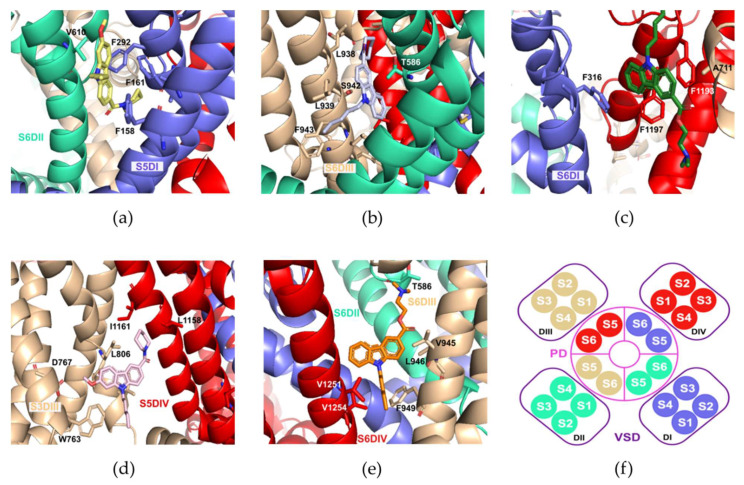
Important interaction residues of the NMP compounds with the Ca_v_3.2 channel, (**a**) NMP-4 in the S6DII site, (**b**) NMP-7 in the S6DIII site, (**c**) NMP-181 in the S6-DIII site, (**d**) NMP-4 in the S5DIV site, and (**e**) NMP-181 in the Pore-blocking site. In (**f**), extracellular view diagram as a reference showing the distribution of the transmembrane segments of the Ca_v_3.2 channel situated in the Pore Domain (PD) and in the Voltage Sensing Domain (VSD).

The results from the molecular docking calculation of the NMP compounds with the Ca_v_3.2 channel suggested distinct possible molecular poses. From these poses, those located at the transmembrane region were further considered, particularly those involving interaction with residues located at the S5 and S6 helical segments (DI-DIV) of the Ca_v_3.2 channel. Five different poses were considered, namely, S6DI, S6DII, S6DIII, S5DIV, and pore-blocking site, Figure 7a–e. Figure 7 shows important residues located in the binding sites of representative NMP compounds with the Ca_v_3.2 channel. It was observed that many hydrophobic and aromatic residues were found in all the binding pockets.

In the S6DII pose (Figure 7a), the carbazole group from the NMP-4 compound interacted with residues F158 and F161 via π-π stacking interactions, and with V610 via π-alkyl interactions. In addition, the F292, I295, and C157 residues interacted with the NMP-4 piperidine group through π-alkyl interactions, in the case of phenylalanine and alkyl-alkyl interactions in the case of isoleucine and cysteine. In the S6DIII pose (Figure 7b), F943 and L939 residues came in close contact with the pentyl group of the NMP-7 compound by alkyl interactions. Further, the L938 residue interacted in a π-alkyl manner with the piperidine ring, while the T586 and S942 residues interacted with the carbazole group in a π-σ and π-hydrogen bond donor, respectively. In addition, for the S6DI pose (Figure 7c), it was observed that the binding pocket was mainly constituted by aromatic phenylalanine residues. The carbazole group in the NMP-181 compound interacted with A711 and F1193 residues with interactions of the π-alkyl and π-π stacking type, respectively, and with residues F316 and F1197 via π-π interactions (specifically a T form stacking). The equivalent S6DI binding site in the recently solved cryo-EM structure of the Ca_v_3.1 channel, exhibited phospholipids and cholesterol molecules in this location [26]. Additionally, it was observed that the equivalent S6DIII site in the Ca_v_1.1 channel played an important role in the interaction with dihydropyridines [27].

In the S5DIV pose (Figure 7d), the methoxy group and pentyl group of the NMP-4 ligand seemed to stabilize its position by forming contacts with residues D767 and W763 located in the segment S3DIII. The piperidine ring on the other hand, interacted with residues L806, L1158, and I1161 through alkyl interactions. Furthermore, the carbazole group interacted with residue I809 residue via π-σ interactions. The X-ray information of the TRPV5 calcium channels with the Econazole ligand showed a corresponding site to the one defined here as S5-DIV for the Ca_v_3.2 channel [28]. Finally in the Pore-blocking site (Figure 7e), a hydrophobic region was formed by residue V945, V952, V1251, V1254, V1255, F949, L946, and L1250. In this hydrophobic environment, the pentyl group and the carbazole group of the NMP-181 ligand form alkyl-alkyl and π-alkyl interactions, respectively. In addition, the amine group of the ligand formed a hydrogen bond interaction with residue T568. From these poses, the one that better described experimental information about the inhibitory function of the NMP ligands is the denominated Pore-blocking site. That is, experimental electrophysiological recordings showed that the blocking of Ca_v_3.2 by the anandamide and NMP compounds modify the inactivation phase of the channel [10,11,12]. It was found that the triad of residues, MFV, in the S6-DIII region contributes to the inactivation phase of the Ca_v_3 [29]. In Ca_v_3.2, residue F949 (the middle residue in the triad) participated in the hydrophobic binding pocket in the pore-blocking site. Further, in the recently solved structure of the cryo-EM Ca_v_3.1 channel with the Z944 ligand, not only a similar binding site was identified for the blocker, but also the ligand displayed interactions with equivalent hydrophobic residues and the T921 polar residue (T586 in Ca_v_3.2 described above) [26]. Lastly, by integrating electrophysiological and computational techniques, a similar pose for the inhibitory action of genistein in the human Ca_v_3.3 calcium channel was identified [30]. Figure 7f shows a diagram of the arrangement of the segments for the four domains that form the Ca_v_3.2 channel.

The lowest energy poses are summarized in the Table 3 for the NMP-4, NMP-7, and NMP-181 compounds. It was observed that the interaction energy value obtained was in the order of 10 kcal/mol, having the NMP-7 compound with the highest interaction energy value. It should be mentioned that further mutagenic studies are necessary to verify the proposed interaction residues.

## 3. Computational Methods

The optimized molecular structures were obtained from BP86 functional [31] and cc-pVTZ basis set [32] in chloroform (non-polar solvent), to simulate a lipidic environment by using implicit solvation model PCM [33]. Initial structures NMP-7 and NMP-181 were taken from the PubChem database [34], while NMP-4 was obtained from NMP-7 by the addition of a methoxy group. ^1^H NMR and ^13^C NMR calculations were carried out by using the GIAO method [35]. In the IR spectroscopy analysis, the VEDA program [36] was used to determine the vibrational modes percentages. Each frequency is shown in terms of the Potential Energy Distribution (10% PED). The scaling factor used was 1.014 [37]. The electronic structure and molecular spectroscopy calculations were performed using the Gaussian16 package [38]. The results are displayed by using the GaussView 6.0 program [39]. Frontier MO, MEP maps, and NBO analyses were realized from optimized molecular structures at BP86/cc-pVTZ theory level. For NBO analysis, the GaussSum program [40] was used to calculate the percentage of molecular orbital contributions.

Global reactivity descriptors, such as chemical potential (μ), electronegativity (χ), hardness (η), softness (s), and electrophilicity index (ω), were evaluated based on conceptual DFT approach [24]. Local reactivity descriptors, such as the Fukui functions f(r) for electrophilic $f^{-}\left(r\right)$ and nucleophilic $f^{+}\left(r\right)$ attacks, and the dual descriptor $f^{\left(2\right)}\left(r\right)$ were determined from the Hirshfeld charges. Hirshfeld population analysis was performed by using the Multiwfn program [41]. In addition, the Parr functions P(r) for electrophilic $P^{-}\left(r\right)$ and nucleophilic $P^{+}\left(r\right)$ attacks were calculated.

The structure of the Ca_v_3.2 channel was obtained by homology modeling using the reported methodology [30]. The amino acid sequence for the Ca_v_3.2 was obtained from the UNIPROT database with accession code O95180|CAC1H_Human. The model obtained from the Modeller 9v10 program [42] was validated with mutagenic experimental studies for important residues known in the channel [43,44,45]. The model contains the amino acids from F80 to A1874 (according to the numbering of the primary sequence), it was omitted the intracellular loops that connect the Domains I-II and the Domain II-III, as well as the carbonyl terminal residues (Figure S2). Molecular docking calculation for Cav3.2 and NMP compounds were made using AutoDockVina (ADV) 1.1.2 [46]. ADV uses a genetic algorithm as a searching method and calculates a binding free energy parameter (-ΔG) as a scoring function. The structure of NMP compounds (NMP-4, NMP-7, and NMP-181) (ligands) were obtained from the optimized structures with BP86/cc-pVTZ method in chloroform. The Cav3.2 (receptor) structure was taken from the homology model described in the previous section. The ligand Gasteiger charges and hydrogen atoms added in the receptor were calculated with Autodock tools 1.5.7rc1 [47], generating PDBQT files for both the ligand and the receptor. The rigid blind docking method was used to find the best ligand-receptor poses, and literature reporting important binding sites on related proteins was used as reference of criteria to discriminate the best poses [26,27,28,29,30]. The ligand-receptor prediction was made using a grid dimension space delimited by a 90 Å × 110 Å × 110 Å box located in center defined by the coordinates: x(21.647), y(22.126), and z(4.242), that contained the entire receptor structure. The search considered the default exhaustiveness value of 8 and 80. For the resulting poses, the default value was utilized, i.e., 10. Docked structures of the lowest energy and different poses were clustered to have a diverse output set to identify possible sites for binding. The interactions were visualized with PyMOL v2.0 [48].

## 4. Conclusions

The NMP compounds structures were obtained as unique structures of minimum energy on the PES. By reproducing accurately ^1^H and ^13^C NMR chemical shifts, the BP86/cc-pVTZ is established as the best-suited level of theory standardizing all our calculations. The minimum energy conformation is similar in the NMP compounds, and they maintain a semi-rigid structure. Calculations were carried out in the chloroform solvent, using the implicit solvation model PCM, to simulate a non-polar environment in the interaction with the Ca_v_3.2 channel. The FMO analysis establishes a ΔE_gap_~3.0 eV for the NMP compounds, with the NMP-4 compound being the most kinetically stable and least reactive according to this parameter. The NMP carbazole group contributed mostly (>75%) to the LUMO orbital. For the HOMO orbital, of NMP-4 and NMP-7, the carbazole group contributed notably in 80% and 86%, respectively, while for NMP-181 the amino group represents 97%. Furthermore, in this analysis, a correlation of gap energy was obtained with respect to logIC_50_ (affinity values for Ca_v_3.2) with an R^2^ of 0.90. The MEP obtained is similar among the NMP compounds with a region of major electronic density charge in the carbonyl group extending to the carbazole group and additionally over the methoxy group of NMP-4 and the amide group in NMP-181. A deficient electronic density charge was observed in the pentyl group. The structural stability, measured by the E (2) parameter, was settled due to the appearance of an electronic delocalization effect located mainly in the electronegative N and O atoms and the carbazole group for the NMP compounds. The global reactivity analysis showed no significant changes in electronegativity, hardness, and electrophilicity index between the NMP compounds. The local reactivity descriptors, Fukui functions, and Parr functions, indicated that the most probable sites to suffer a nucleophilic attack were the C21, N17, and N6 atoms for NMP-4, NMP-7, and NMP-181, respectively. According to the Fukui function, the most probable zone to electrophilic attack was C20 for NMP-7 and C13 for NMP-4 and NMP-181. In this respect, the Parr function indicated C13 as the most likely for the electrophilic attack in the NMP compounds. In summary, the main functional group in the NMP compounds was the carbazole group, according to the HOMO-LUMO orbital distributions, molecular electrostatic potential distribution, and local reactivity Fukui indices. The last conclusion indicated that this molecular region was the most probable for undergoing nucleophilic and electrophilic attacks and could be an important region during the ligand-receptor interaction process with the T-type calcium channels and the CB1/CB2 receptors. A molecular docking helped to determine important amino acids of interaction, being the site of the pore-blocking, a probable binding pocket for the compounds NMP in interaction with Ca_v_3.2 channel.

## Figures and Tables

**Table 1:** Selected bond lengths (Å), valence angles, and dihedral angles (degrees) for the NMP compounds at the BP86/cc-pVTZ level of theory in chloroform.

Parameter	NMP-4	NMP-7	NMP-181
O1-C2	1.24	1.24	1.23
N3-C2	1.38	1.38	-
O3-C2	-	-	1.36
N3-C4	1.47	1.47	-
O3-C4	-	-	1.45
N17-C22	1.46	1.46	1.46
O1-C2-N3	122	122	-
O1-C2-O3	-	-	123
C2-N3-C4	119	119	-
C2-O3-C4	-	-	115
O1-C2-C9	120	119	125
C2-C9-C10	118	117	118
C22-C23-C24	112	112	112
C28-O27-C20	118	-	-
C6-C7-C8	111	111	-
O3-C4-C5	-	-	107
O1-C2-N3-C4	−160	−161	-
O1-C2-O3-C4	-	-	0
C5-C4-N3-C2	−138	−136	-
C5-C4-O3-C2	-	-	174
C9-C2-N3-C4	22	21	-
C9-C2-O3-C4	-	-	−180
C23-C24-C25-C26	180	180	−179
C19-C20-O27-C28	0	-	-
C6-C7-C8-N3	54	55	-
O3-C4-C5-N6	-	-	179

**Table 2:** Global reactivity descriptors (eV) for NMP compounds at BP86/cc-pVTZ level of theory in chloroform.

	NMP-4	NMP-7	NMP-181
µ	−3.44	−3.57	−3.62
χ	3.44	3.57	3.62
η	3.17	3.14	3.00
s	0.32	0.32	0.33
ω	1.87	2.02	2.19

**Table 3:** ΔG (kcal/mol) interaction energy of the anandamide conformers and NMP compounds with the Ca_v_3.2 channel.

Sites	NMP-4	NMP-7	NMP-181
S6DI	−9.1	−10.0	−9.6
S6DII	−9.5	−10.0	−9.1
S6DIII	−9.5	−10.1	−9.2
S5DIV	−7.9	−8.1	−8.2
Pore-blocking	−9.4	−9.1	−8.2

## Data Availability

Not applicable.

## Supplementary Materials

The following are available online. Table S1: experimental and calculated ^1^H NMR δ (ppm) at BP86/cc-pVTZ level of theory in chloroform for NMP compounds; Table S2: Experimental and calculated ^13^C NMR δ (ppm) at BP86/cc-pVTZ level of theory in chloroform for NMP compounds; Table S3: theoretical IR frequencies (in cm^−1^) and PED (≥10%) at BP86/cc-pVTZ level of theory in chloroform for NMP compounds using the scale factor of 1.014. ν refers to stretching vibrational mode, β refers to in plane bending vibrational mode, and τ refers to torsional vibrational mode; Table S4: selected bond lengths (Å), valence angles and dihedral angles (degrees) for the NMP compounds at BP86/cc-pVTZ level of theory in chloroform solvent; Table S5: molecular orbital contribution percentages of the NMP compounds at BP86/cc-pVTZ level of theory in chloroform; Table S6: NBO analysis (donor→acceptor) for NMP compounds at BP86/cc-pVTZ level of theory in chloroform; Table S7: Fukui functions, $f^{+}\left(r\right)$ and $f^{-}\left(r\right)$, dual descriptor, $f^{\left(2\right)}\left(r\right)$, and Parr functions, $P^{-}\left(r\right)$ and $P^{+}\left(r\right)$, for the NMP compounds at BP86/cc-pVTZ level of theory in chloroform; Figure S1: correlation graph ΔE_gap_ values (in eV) with the experimental logIC_50_ values (in µM) for the NMP compounds obtained at BP86/cc-pVTZ level of theory in chloroform solvent; Figure S2: (**A**) diagram of the structure Ca_v_3.2 channel, in color code represented the four domains: the domain I (DI) in blue, the domain II (DII) in green, the domain III (DIII) in beige, and the domain IV (DIV) in red. Each domain contains six transmembrane segments (S1–S6). The segments S1–S4 form the voltage-sensing domain (VSD) and the segments S5–S6 with the segment P (P1 and P2) form the pore domain (PD). (**B**) Lateral view of the human Ca_v_3.2 channel generated by homology modeling, the extracellular (EC), transmembrane (TM) and intracellular (IC) portion are indicated. (**C**) Extracellular view of the human Ca_v_3.2 channel. (**D**) Extracellular view diagram of the spatial distribution of the transmembrane segments of the human Ca_v_3.2 channel.

## References

[1] Wu J. (2019). Cannabis, cannabinoid receptors, and endocannabinoid system: Yesterday, today, and tomorrow. Acta Pharmacol. Sin..

[2] Pacher P., Bátkai S., Kunos G. (2006). The Endocannabinoid System as an Emerging Target of Pharmacotherapy. Pharmacol. Rev..

[3] Colino L., Herranz-Herrer J., Gil-Benito E., Ponte-Lopez T., del Sol-Calderon P., Rodrigo-Yanguas M., Gil-Ligero M., Sánchez-López A.J., de Leon J., Blasco-Fontecilla H. (2018). Cannabinoid Receptors, Mental Pain and Suicidal Behavior: A Systematic Review. Curr. Psychiatry Rep..

[4] Todorovic S.M., Jevtovic-Todorovic V. (2013). Neuropathic pain: Role for presynaptic T-type channels in nociceptive signaling. Pflugers Arch. Eur. J. Physiol..

[5] Tibbs G.R., Posson D.J., Goldstein P.A. (2016). Voltage-Gated Ion Channels in the PNS: Novel Therapies for Neuropathic Pain?. Trends Pharmacol. Sci..

[6] Snutch T.P., Zamponi G.W. (2018). Recent advances in the development of T-type calcium channel blockers for pain intervention. Br. J. Pharmacol..

[7] Yang Y., Cui Y., Sang K., Dong Y., Ni Z., Ma S., Hu H. (2018). Ketamine blocks bursting in the lateral habenula to rapidly relieve depression. Nature.

[8] Knölker H.J., Reddy K.R. (2002). Isolation and synthesis of biologically active carbazole alkaloids. Chem. Rev..

[9] Bashir M., Bano A., Ijaz A.S., Chaudhary B.A. (2015). Recent developments and biological activities of n-substituted carbazole derivatives: A review. Molecules.

[10] Gadotti V.M., You H., Petrov R.R., Berger N.D., Diaz P., Zamponi G.W. (2013). Analgesic Effect of a Mixed T-Type Channel Inhibitor/CB2 Receptor Agonist. Mol. Pain.

[11] You H., Gadotti V.M., Petrov R.R., Zamponi G.W., Diaz P. (2011). Functional characterization and analgesic effects of mixed cannabinoid receptor/T-type channel ligands. Mol. Pain.

[12] Berger N.D., Gadotti V.M., Petrov R.R., Chapman K., Diaz P., Zamponi G.W. (2014). NMP-7 inhibits chronic inflammatory and neuropathic pain via block of Ca_v_3.2 T-type calcium channels and activation of CB2 receptors. Mol. Pain.

[13] Silverstein R., Webster F. (2005). Spectrometric Identification of Organic Compounds.

[14] Pavia D., Lampman G., Kriz G. (2001). Introduction to Spectroscopy: A Guide for Students of Organic Chemistry.

[15] Socrates G. (2004). Infrared and Raman Characteristic Group Frequencies: Tables and Charts.

[16] Abu-Dief A.M., El-khatib R.M., Aljohani F.S., Alzahrani S.O., Mahran A., Khalifa M.E., El-Metwaly N.M. (2021). Synthesis and intensive characterization for novel Zn(II), Pd(II), Cr(III) and VO(II)-Schiff base complexes; DNA-interaction, DFT, drug-likeness and molecular docking studies. J. Mol. Struct..

[17] Abdel-Rahman L.H., Adam M.S., Abu-Dief A.M., Ahmed H.E.S., Nafady A. (2020). Non-Linear Optical Property and Biological Assays of Therapeutic Potentials Under In Vitro Conditions of Pd(II), Ag(I) and Cu(II) Complexes of 5-Diethyl amino-2-({2-[(2-hydroxy-Benzylidene)-amino]-phenylimino}-methyl)-phenol. Molecules.

[18] Abdel-Rahman L.H., Abu-Dief A.M., Shehata M.R., Atlam F.M., Abdel-Mawgoud A.A.H. (2019). Some new Ag(I), VO(II) and Pd(II) chelates incorporating tridentate imine ligand: Design, synthesis, structure elucidation, density functional theory calculations for DNA interaction, antimicrobial and anticancer activities and molecular docking studies. Appl. Organomet. Chem..

[19] Jensen F. (2007). Introduction to Computational Chemistry.

[20] Serdaroğlu G., Ortiz J.V. (2017). Ab Initio Calculations on some Antiepileptic Drugs such as Phenytoin, Phenbarbital, Ethosuximide and Carbamazepine. Struct. Chem..

[21] Bladen C., McDaniel S.W., Gadotti V.M., Petrov R.R., Berger N.D., Diaz P., Zamponi G.W. (2015). Characterization of novel cannabinoid based T-type calcium channel blockers with analgesic effects. ACS Chem. Neurosci..

[22] Gómez-Jeria J.S. (2014). A quantum-chemical analysis of the relationships between hCB2 cannabinoid receptor binding affinity and electronic structure in a family of 4-oxo-1,4-dihydroquinoline-3-carboxamide derivatives. Pharm. Lett..

[23] Gangadharana R.P., Krishnanb S.S. (2014). Natural Bond Orbital (NBO) population analysis of 1-azanapthalene-8-ol. Acta Phys. Pol. A.

[24] Domingo L.R., Ríos-Gutiérrez M., Pérez P. (2016). Applications of the conceptual density functional theory indices to organic chemistry reactivity. Molecules.

[25] Parthasarathi R., Subramanian V., Roy D.R., Chattaraj P.K. (2004). Electrophilicity index as a possible descriptor of biological activity. Bioorg. Med. Chem..

[26] Zhao Y., Huang G., Wu Q., Wu K., Li R., Lei J., Pan X., Yan N. (2019). Cryo-EM structures of apo and antagonist-bound human Ca_v_3.1. Nature.

[27] Mitterdorfer J., Grabner M., Kraus R.L., Hering S., Prinz H., Glossmann H., Striessnig J. (1998). Molecular basis of drug interaction with L-type Ca^2+^ channels. J. Bioenerg. Biomembr..

[28] Hughes T.E.T., Pumroy R.A., Yazici A.T., Kasimova M.A., Fluck E.C., Huynh K.W., Samanta A., Molugu S.K., Zhou Z.H., Carnevale V. (2018). Structural insights on TRPV5 gating by endogenous modulators. Nat. Commun..

[29] Marksteiner R., Schurr P., Berjukow S., Margreiter E., Perez-Reyes E., Hering S. (2001). Inactivation determinants in segment IIIS6 of Ca_v_3.1. J. Physiol..

[30] Rangel-Galván M., Rangel A., Romero-Méndez C., Dávila E.M., Castro M.E., Caballero N.A., Meléndez Bustamante F.J., Sanchez-Gaytan B.L., Meza U., Perez-Aguilar J.M. (2021). Inhibitory Mechanism of the Isoflavone Derivative Genistein in the Human Ca_v_3.3 Channel. ACS Chem. Neurosci..

[31] Becke A.D. (1988). Density-functional exchange-energy approximation with correct asymptotic behavior. Phys. Rev. A.

[32] Dunning T.H. (1989). Gaussian basis sets for use in correlated molecular calculations. I. The atoms boron through neon and hydrogen. J. Chem. Phys..

[33] Tomasi J., Mennucci B., Cammi R. (2005). Quantum mechanical continuum solvation models. Chem. Rev..

[34] Kim S., Thiessen P.A., Bolton E.E., Chen J., Fu G., Gindulyte A., Han L., He J., He S., Shoemaker B.A. (2016). PubChem substance and compound databases. Nucleic Acids Res..

[35] Wolinski K., Hinton J.F., Pulay P. (1990). Efficient Implementation of the Gauge-Independent Atomic Orbital Method for NMR Chemical Shift Calculations. J. Am. Chem. Soc..

[36] Jamróz M.H. (2013). Vibrational energy distribution analysis (VEDA): Scopes and limitations. Spectrochim. Acta—Part A Mol. Biomol. Spectrosc..

[37] Alecu I.M., Zheng J., Zhao Y., Truhlar D.G. (2010). Computational thermochemistry: Scale factor databases and scale factors for vibrational frequencies obtained from electronic model chemistries. J. Chem. Theory Comput..

[38] Frisch M.J., Trucks G.W., Schlegel H.B., Scuseria G.E., Robb M.A., Cheeseman J.R., Scalmani G., Barone V., Petersson G.A., Nakatsuji H. (2016). Gaussian 16, Revision B.O1.

[39] Dennington R., Tood A.K., Jhon M.M. (2016). GaussView.

[40] O’boyle N.M., Tenderholt A.L., Langner K.M. (2008). cclib: A library for package-independent computational chemistry algorithms. J. Comput. Chem..

[41] Lu T., Chen F. (2012). Multiwfn: A multifunctional wavefunction analyzer. J. Comput. Chem..

[42] Sali A., Blundell T.L. (1993). Comparative protein modeling by satisfaction of spatial restraints. J. Mol. Biol..

[43] Khosravani H., Bladen C., Parker D.B., Snutch T.P., McRory J.E., Zamponi G.W. (2005). Effects of Ca_v_3.2 channel mutations linked to idiopathic generalized epilepsy. Ann. Neurol..

[44] Peloquin J.B., Khosravani H., Barr W., Bladen C., Evans R., Mezeyova J., Parker D., Snutch T.P., McRory J.E., Zamponi G.W. (2006). Functional analysis of Ca_v_3.2 T-type calcium channel mutations linked to childhood absence epilepsy. Epilepsia.

[45] Arias-Olguín I.I., Vitko I., Fortuna M., Baumgart J.P., Sokolova S., Shumilin I.A., Van Deusen A., Soriano-García M., Gomora J.C., Perez-Reyes E. (2008). Characterization of the gating brake in the I-II loop of Ca_v_3.2 T-type Ca^2+^ channels. J. Biol. Chem..

[46] Trott O., Olson A.J. (2010). Autodock vina: Improving the speed and accuracy of docking. J. Comput. Chem..

[47] Morris G.M., Huey R., Lindstrom W., Sanner M.F., Belew R.K., Goodsell D.S., Olson A.J. (2009). AutoDock4 and AutoDockTools4: Automated docking with selective receptor flexibility. J. Comput. Chem..

[48] Schrödinger L. (2017). The PyMOL Molecular Graphics System.

