# Supplementary material for: Theoretical Study of the Structural Stability, Chemical Reactivity, and Protein Interaction for NMP Compounds as Modulators of the Endocannabinoid System
Source: Molecules. 2022 Jan 9;27(2):414. doi: 10.3390/molecules27020414 (PMC8779749; doi:10.3390/molecules27020414)
Supplement: Supplementary file 1 [file molecules-27-00414-s001.zip › molecules-1479268-supplementary.pdf]

# Supplementary Materials

## Theoretical study of the structural stability, chemical reactivity, and protein interaction for NMP compounds as modulators of the endocannabinoid system.

**Maricruz Rangel-Galván [1], María Eugenia Castro [2,*], Jose Manuel Perez-Aguilar[1], Norma A. Caballero[3], Alejandro Rangel-Huerta[4], Francisco Javier Meléndez [2,*]**

[1] Lab. de Química Teórica, Centro de Investigación, Depto. de Fisicoquímica, Facultad de Ciencias Químicas, Benemérita Universidad Autónoma de Puebla, Edif. FCQ10, 22 Sur y San Claudio, Ciudad Universitaria, Col. San Manuel, C.P 72570. Puebla, Puebla, México; maricruz.rangel@alumno.buap.mx; jmanuel.perez@correo.buap.mx; francisco.melendez@correo.buap.mx

[2] Centro de Química, Instituto de Ciencias, Benemérita Universidad Autónoma de Puebla, Complejo de Ciencias, ICUAP, Edif. IC8, 22 Sur y San Claudio, Ciudad Universitaria, Col. San Manuel, C.P 72570. Puebla, Puebla, México; mareug.castro@correo.buap.mx

[3] Facultad de Ciencias Biológicas, Benemérita Universidad Autónoma de Puebla, Edif. BIO1, 22 Sur y San Claudio, Ciudad Universitaria, Col. San Manuel, C.P 72570. Puebla, Puebla, México; norma.caballero@correo.buap.mx

[4] Facultad de Ciencias de la Computación, Benemérita Universidad Autónoma de Puebla, Edif. CCO2, 22 Sur y San Claudio, Ciudad Universitaria, Col. San Manuel, C.P 72570. Puebla, Puebla, México; alejandro.rangel@correo.buap.mx

* Correspondence: mareug.castro@correo.buap.mx; francisco.melendez@correo.buap.mx; Tel.: (2222295500 MEC ext 2819; FJM ext 2830)

**Table S1.** Experimental and calculated $^{1}$H NMR δ (ppm) at BP86/cc-pVTZ level of theory in chloroform for NMP compounds.

| | Groups | Exp. [9, 10] | $CHCl_3$ |
|---|---|---|---|
| **NMP-4** | $H_{ring}$ | 6.86-8.07 | 6.98-8.18 |
| | $H_{CH3-O}$ | 3.93 | 3.89-4.11 |
| | $H_{R-CH2-N}$ | 4.22 | 4.24-4.37 |
| | $H_{R-CH2-N*}$ | 3.71 | 2.55-4.97 |
| | $H_{R-CH2-R'**}$ | 1.62-1.69 | 1.50-1.99 |
| | $H_{CH2}$ | 1.35-1.85 | 1.40-1.76 |
| | $H_{CH3}$ | 0.88 | 0.94-1.21 |
| | **$R^2$** | | 0.9740 |
| **NMP-7** | $H_{ring}$ | 7.23-8.19 | 7.51-8.33 |
| | $H_{R-CH2-N}$ | 4.25 | 4.42-4.43 |
| | $H_{R-CH2-N*}$ | 3.73 | 2.60-5.05 |
| | $H_{R-CH2-R'**}$ | 1.61-1.67 | 1.60-2.07 |
| | $H_{CH2}$ | 1.32-1.83 | 1.40-1.83 |
| | $H_{CH3}$ | 0.85 | 0.94-1.22 |
| | **$R^2$** | | 0.9769 |
| **NMP-181** | $H_{ring}$ | 7.23-8.81 | 7.56-9.00 |
| | $H_{CH2-O}$ | 4.48 | 4.15-4.71 |
| | $H_{R-CH2-N}$ | 4.21 | 4.42-4.43 |
| | $H_{CH2-N}$ | 2.76 | 2.52-3.17 |
| | $H_{CH3-N}$ | 2.36 | 1.73-2.83 |
| | $H_{CH2}$ | 1.26-1.81 | 1.44-1.87 |
| | $H_{CH3}$ | 0.84 | 0.93-1.21 |
| | **$R^2$** | | 0.9928 |

*cicloalkane; **cyclic

**Table S2.** Experimental and calculated $^{13}C$ NMR δ (ppm) at BP86/cc-pVTZ level of theory in chloroform for NMP compounds.

| | Groups | Exp. [10,11] | $CHCl_3$ |
|---|---|---|---|
| **NMP-4** | $C_{C=O}$ | 171.7 | 174.2 |
| | $C_{C-OCH3}$ | 159.5 | 164.4 |
| | $C_{C=N-R}$ | 141.3-142.4 | 143.8-144.8 |
| | $C_{ring}$ | 119-127 | 121.0-131.0 |
| | $C_{CH2ring}$ | 93.6 -121.4 | 96.9-128.3 |
| | $C_{CH3-O}$ | | 58.5 |
| | $C_{CH2-C}$* | 24.9-28.6 | 29.4-31.1 |
| | $C_{CH2-N}$* | 43.3-55.8 | 47.3-54.7 |
| | $C_{CH2-N}$ | | 47.4 |
| | $C_{CH2-C}$ | 22.6 - 29.5 | 29.4-34.9 |
| | $C_{CH3-C}$ | 14.11 | 16.5 |
| | **$R^2$** | | 0.9977 |
| **NMP-7** | $C_{C=O}$ | 171.5 | 173.2 |
| | $C_{C=N-R}$ | 140.9-141.0 | 143.0-143.5 |
| | $C_{ring}$ | 125.1-126.7 | 124.1-131.1 |
| | $C_{H2ring}$ | 108.4-122.4 | 110.0-130.4 |
| | $C_{CH2-C}$* | 29.4 | 30.0-31.0 |
| | $C_{CH2-N}$* | | 47.4-54.0 |
| | $C_{CH2-N}$ | 43.2 | 48.1 |
| | $C_{CH2-C}$ | 22.5-28.7 | 29.5-35.1 |
| | $C_{CH3-C}$ | 14.0 | 16.6 |
| | **$R^2$** | | 0.9799 |
| **NMP-181** | $C_{C=O}$ | 167.5 | 171.3 |
| | $C_{C=N-R}$ | 141.0-143.1 | 143.8-145.0 |
| | $C_{ring}$ | 120.7-122.6 | 123.6-126.5 |
| | $C_{H2ring}$ | 108.2-127.4 | 109.7-130.2 |
| | $C_{CH2-O}$ | 58.1 | 69.0 |
| | $C_{CH3-N}$ | 46.04 | 47.0-51.7 |
| | $C_{CH2-N}$ | 43.3-62.9 | 48.5-62.8 |
| | $C_{CH2-C}$ | 22.5-29.4 | 29.5-35.3 |
| | $C_{CH3-C}$ | 14.0 | 16.6 |
| | **$R^2$** | | 0.9732 |

**Table S3.** Theoretical IR frequencies (in cm-1) and PED (≥10%) at BP86/cc-pVTZ level of theory in chloroform for NMP compounds using the scale factor of 1.014. ν refers to stretching vibrational mode, β refers to in plane bending vibrational mode, and τ refers to torsional vibrational mode.

| | Freq | Int | Freq esc | PED (≥ 10%) |
|---|---|---|---|---|
| **NMP-4** | 3063.1 | 6.07 | 3106.0 | νCH(91) |
| | 3012.2 | 22.23 | 3054.3 | νCH(86) |
| | 2997.4 | 22.19 | 3039.3 | νCH(91) |
| | 2949.7 | 9.33 | 2991.0 | νCH(90) |
| | 2939.3 | 9.79 | 2980.5 | νCH(86) |
| | 2935.4 | 24.78 | 2976.5 | νCH(91) |
| | 1612.4 | 55.73 | 1635.0 | νCC(50) |
| | 1600.8 | 88.20 | 1623.2 | νOC(77) |
| | 1221.9 | 29.54 | 1239.0 | νOC(13) |
| | 577.0 | 12.69 | 585.0 | βOCC(13) |
| **NMP-7** | 3118.7 | 7.41 | 3162.3 | νCH(93) |
| | 3013.5 | 25.38 | 3055.7 | νCH(93) |
| | 2997.3 | 23.28 | 3039.3 | νCH(83) |
| | 2951.5 | 10.61 | 2992.8 | νCH(90) |
| | 2947.7 | 10.72 | 2989.0 | νCH(88) |
| | 2939.8 | 10.99 | 2980.9 | νCH(86) |
| | 1607.0 | 17.17 | 1629.5 | νCC(33) |
| | 1597.2 | 100.00 | 1619.5 | νOC(75) |
| | 1396.9 | 98.81 | 1416.5 | νNC(29)+βHCN(32) |
| | 1331.3 | 37.90 | 1349.9 | νNC(26) |
| **NMP-181** | 3119.9 | 4.91 | 3163.6 | νCH(93) |
| | 3036.8 | 6.81 | 3079.3 | νCH(94) |
| | 3013.4 | 12.14 | 3055.6 | νCH(75) |
| | 2939.9 | 7.52 | 2981.0 | νCH(87) |
| | 2836.9 | 49.51 | 2876.6 | νCH(98) |
| | 1668.8 | 100.00 | 1692.1 | νOC(85) |
| | 1606.9 | 14.75 | 1629.4 | νCC(39) |
| | 1246.3 | 4.43 | 1263.7 | νNC(15) + τHCNC(31) |
| | 1190.5 | 80.04 | 1207.1 | νOC(11)+νCC(12)+βCCC(12) |
| | 1073.5 | 47.18 | 1088.5 | νOC(24) |

**Table S4.** Selected bond lengths (Å), valence angles and dihedral angles (degrees) for the NMP compounds at BP86/cc-pVTZ level of theory in chloroform solvent.

| Compound | $E_{HOMO}$ | $E_{LUMO}$ | $\Delta E_{gap}$ |
|---|---|---|---|
| **NMP-4** | -5.024 | -1.855 | 3.169 |
| **NMP-7** | -5.137 | -1.996 | 3.142 |
| **NMP-181** | -5.125 | -2.122 | 3.002 |

**Table S5.** Molecular orbital contribution percentages of the NMP compounds at BP86/cc-pVTZ level of theory in chloroform.

| | MO | | eV | Carbazole | Pentyl | Carbonyl | Piperidine | Methoxy |
|---|---|---|---|---|---|---|---|---|
| | 106 | L+3 | -0.16 | 82 | 4 | 7 | 4 | 3 |
| | 105 | L+2 | -0.79 | 85 | 2 | 9 | 3 | 1 |
| | 104 | L+1 | -1.49 | 70 | 0 | 22 | 8 | 0 |
| | 103 | LUMO | -1.85 | 95 | 1 | 1 | 1 | 3 |
| **NMP-4** | 102 | HOMO | -5.02 | 80 | 3 | 8 | 1 | 9 |
| | 101 | H-1 | -5.19 | 89 | 3 | 0 | 0 | 8 |
| | 100 | H-2 | -5.57 | 6 | 0 | 46 | 47 | 1 |
| | 99 | H-3 | -5.86 | 18 | 0 | 50 | 30 | 2 |
| | MO | | eV | Carbazole | Pentyl | Carbonyl | Piperidine | |
| | 98 | L+3 | -0.37 | 92 | 2 | 3 | 2 | |
| | 97 | L+2 | -0.82 | 88 | 2 | 7 | 3 | |
| | 96 | L+1 | -1.55 | 70 | 1 | 22 | 8 | |
| | 95 | LUMO | -2.00 | 97 | 1 | 1 | 1 | |
| **NMP-7** | 94 | HOMO | -5.14 | 86 | 5 | 7 | 2 | |
| | 93 | H-1 | -5.5 | 65 | 0 | 27 | 8 | |
| | 92 | H-2 | -5.64 | 26 | 0 | 23 | 51 | |
| | 91 | H-3 | -5.94 | 30 | 0 | 50 | 19 | |
| | MO | | eV | Carbazole | Pentyl | Ester | Amine | |
| | 99 | L+3 | -0.43 | 95 | 3 | 2 | 0 | |
| | 98 | L+2 | -0.92 | 94 | 2 | 4 | 0 | |
| | 97 | L+1 | -1.99 | 83 | 1 | 16 | 0 | |
| | 96 | LUMO | -2.12 | 76 | 0 | 23 | 0 | |
| **NMP-181** | 95 | HOMO | -5.12 | 0 | 0 | 2 | 97 | |
| | 94 | H-1 | -5.29 | 90 | 6 | 4 | 0 | |
| | 93 | H-2 | -5.69 | 98 | 0 | 1 | 0 | |
| | 92 | H-3 | -6.43 | 9 | 0 | 90 | 1 | |

**Table S6.** NBO analysis (donor→acceptor) for NMP compounds at BP86/cc-pVTZ level of theory in chloroform. The numbering of the atoms corresponds to Figure 1.

| | Donnor NBO (i) | Acceptor NBO (j) | E(2)[a] (kcal $mol^{-1}$) | E(j)-E(i)[b] (a.u.) | F(i,j)[c] (a.u.) |
|---|---|---|---|---|---|
| **NMP-4** | π ($C_9$-$C_{13}$) | π* ($O_1$-$C_2$) | 11.10 | 0.29 | 0.051 |
| | π ($C_9$-$C_{13}$) | π* ($C_{10}$-$C_{11}$) | 15.70 | 0.23 | 0.054 |
| | π ($C_{10}$-$C_{11}$) | π* ($C_9$-$C_{13}$) | 13.00 | 0.23 | 0.050 |
| | LP ($O_1$) | π* ($C_2$-$N_3$) | 20.28 | 0.59 | 0.099 |
| | LP ($O_1$) | π* ($C_2$-$C_9$) | 16.61 | 0.55 | 0.087 |
| | LP ($N_3$) | π* ($O_1$-$C_2$) | 25.75 | 0.28 | 0.077 |
| | LP ($N_{17}$) | π* ($C_{16}$-$C_{21}$) | 38.69 | 0.22 | 0.084 |
| | LP ($O_{27}$) | π* ($C_{20}$-$C_{19}$) | 27.36 | 0.26 | 0.081 |
| | π* ($C_9$-$C_{13}$) | π* ($O_1$-$C_2$) | 23.28 | 0.06 | 0.056 |
| | π* ($O_1$-$C_2$) | π* ($O_1$-$C_2$) | 23.91 | 0.36 | 0.190 |
| **NMP-7** | π ($C_9$-$C_{13}$) | π* ($O_1$-$C_2$) | 11.61 | 0.29 | 0.052 |
| | π ($C_9$-$C_{13}$) | π* ($C_{10}$-$C_{11}$) | 15.81 | 0.23 | 0.054 |
| | π ($C_{10}$-$C_{11}$) | π* ($C_9$-$C_{13}$) | 12.73 | 0.23 | 0.050 |
| | π ($C_{15}$-$C_{18}$) | π* ($C_{16}$-$C_{21}$) | 16.02 | 0.22 | 0.054 |
| | LP ($O_1$) | π* ($C_2$-$N_3$) | 20.24 | 0.59 | 0.099 |
| | LP ($O_1$) | π* ($C_2$-$C_9$) | 16.59 | 0.55 | 0.087 |
| | LP ($N_3$) | π* ($O_1$-$C_2$) | 26.51 | 0.28 | 0.078 |
| | π* ($O_1$-$C_2$) | π* ($O_1$-$C_2$) | 24.22 | 0.36 | 0.190 |
| | π* ($C_{12}$-$N_{17}$) | π* ($C_{10}$-$C_{11}$) | 83.57 | 0.05 | 0.077 |
| | π* ($C_9$-$C_{13}$) | π* ($O_1$-$C_2$) | 23.42 | 0.06 | 0.058 |
| **NMP-181** | π ($C_9$-$C_{13}$) | π* ($O_1$-$C_2$) | 23.59 | 0.2 | 0.061 |
| | π ($C_9$-$C_{13}$) | π* ($C_{10}$-$C_{11}$) | 16.61 | 0.23 | 0.055 |
| | π ($C_{10}$-$C_{11}$) | π* ($C_9$-$C_{13}$) | 11.98 | 0.23 | 0.048 |
| | π ($C_{14}$-$C_{12}$) | π* ($C_{15}$-$C_{16}$) | 16.23 | 0.22 | 0.054 |
| | LP ($O_1$) | π* ($C_2$-$O_3$) | 28.07 | 0.51 | 0.108 |
| | LP ($O_1$) | π* ($C_2$-$C_9$) | 16.45 | 0.58 | 0.089 |
| | LP ($O_3$) | π* ($O_1$-$C_2$) | 40.21 | 0.25 | 0.091 |
| | LP ($N_{17}$) | π* ($C_{14}$-$C_{12}$) | 33.81 | 0.22 | 0.079 |
| | LP ($N_6$) | σ* ($C_8$-$H_{53}$) | 6.99 | 0.55 | 0.056 |
| | π* ($O_1$-$C_2$) | π* ($C_9$-$C_{13}$) | 62.65 | 0.03 | 0.065 |

[a] E(2) is the stabilizing energy

[b] E(j)-E(i) es the energy difference between NBO donor (i) and acceptor (j) orbitals

[c] F(i,j) are the Fock matrix elements between NBO donor (i) and acceptor (j) orbitals

**Table S7.** Fukui functions, $f^+(r)$ and $f^-(r)$, dual descriptor, $f^{(2)}(r)$, and Parr functions, $P^-(r)$ and $P^+(r)$, for the NMP compounds at BP86/cc-pVTZ level of theory in chloroform.

| | | $\boldsymbol{f^+(r)}$ | $\boldsymbol{f^-(r)}$ | $\boldsymbol{f^{(2)}(r)}$ | $\boldsymbol{P^+(r)}$ | $\boldsymbol{P^-(r)}$ |
|---|---|---|---|---|---|---|
| **NMP-4** | O1 | 0.063 | 0.023 | 0.040 | 0.104 | 0.003 |
| | C9 | 0.049 | 0.029 | 0.020 | 0.107 | 0.017 |
| | C10 | 0.041 | 0.063 | -0.022 | 0.034 | 0.097 |
| | C11 | 0.037 | 0.064 | -0.026 | 0.033 | 0.089 |
| | C13 | 0.025 | 0.070 | -0.045 | -0.006 | 0.157 |
| | C14 | 0.043 | 0.044 | -0.002 | 0.084 | 0.088 |
| | N17 | 0.050 | 0.011 | 0.038 | 0.129 | 0.001 |
| | C20 | 0.040 | 0.053 | -0.013 | 0.055 | 0.095 |
| | C21 | 0.072 | 0.052 | 0.020 | 0.146 | 0.055 |
| | O27 | 0.048 | 0.027 | 0.021 | 0.079 | 0.022 |
| **NMP-7** | O1 | 0.060 | 0.025 | 0.035 | 0.089 | 0.006 |
| | C9 | 0.054 | 0.031 | 0.022 | 0.117 | 0.020 |
| | C10 | 0.031 | 0.061 | -0.030 | -0.007 | 0.092 |
| | C11 | 0.049 | 0.064 | -0.015 | 0.074 | 0.088 |
| | C13 | 0.035 | 0.073 | -0.037 | 0.029 | 0.167 |
| | N17 | 0.072 | 0.012 | 0.060 | 0.205 | 0.003 |
| | C18 | 0.046 | 0.067 | -0.022 | 0.045 | 0.121 |
| | C19 | 0.062 | 0.041 | 0.021 | 0.093 | 0.009 |
| | C20 | 0.036 | 0.074 | -0.038 | -0.002 | 0.122 |
| | C21 | 0.055 | 0.059 | -0.004 | 0.095 | 0.072 |
| **NMP-181** | O1 | 0.028 | 0.061 | -0.033 | 0.018 | 0.059 |
| | C2 | 0.014 | 0.048 | -0.034 | 0.006 | 0.077 |
| | N6 | 0.086 | 0.002 | 0.085 | 0.204 | 0.000 |
| | C11 | 0.039 | 0.057 | -0.018 | 0.066 | 0.061 |
| | C13 | 0.024 | 0.100 | -0.076 | 0.015 | 0.248 |
| | N17 | 0.054 | 0.014 | 0.041 | 0.157 | 0.003 |
| | C18 | 0.033 | 0.051 | -0.018 | 0.028 | 0.078 |
| | C19 | 0.050 | 0.036 | 0.014 | 0.084 | 0.008 |
| | C20 | 0.027 | 0.064 | -0.037 | -0.004 | 0.097 |
| | C21 | 0.042 | 0.044 | -0.002 | 0.074 | 0.040 |

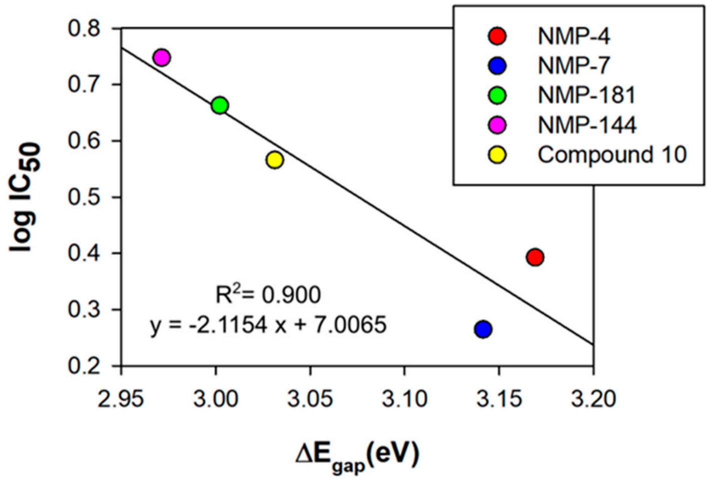


**Figure S1.** Correlation graph $\Delta E_{gap}$ values (in eV) with the experimental logIC50 values (in μM) for the NMP compounds obtained at BP86/cc-pVTZ level of theory in chloroform solvent.

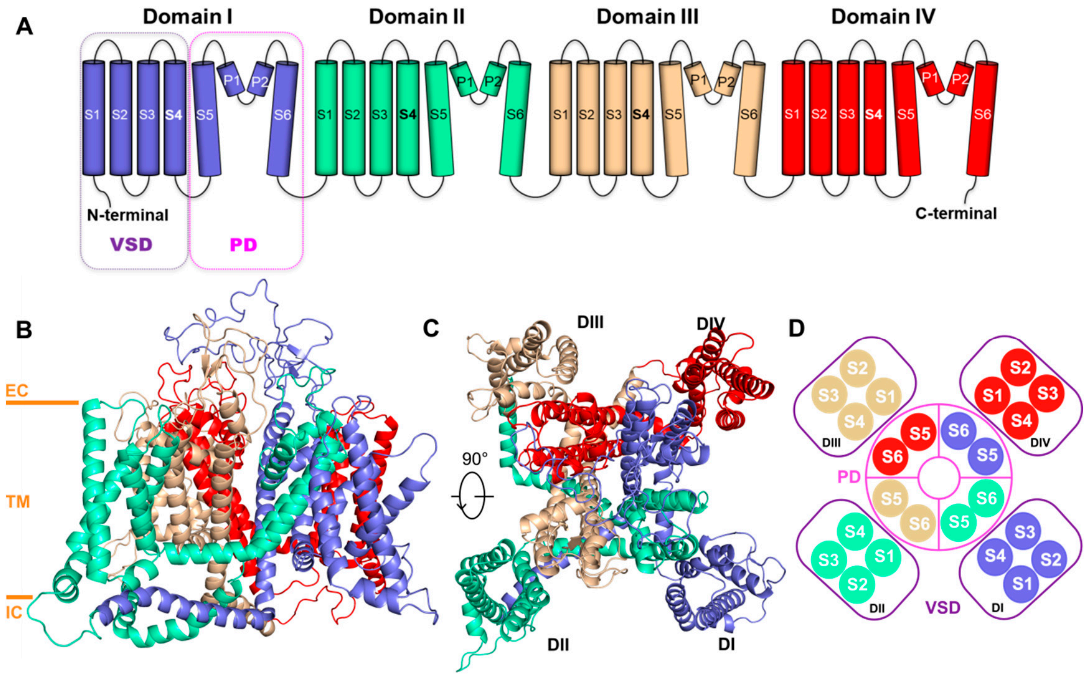


**Figure S2.** (A) Diagram of the structure Cav3.2 channel, in color code represented the four domains: the Domain I (DI) in blue, the Domain II (DII) in green, the Domain III (DIII) in beige, and the Domain IV (DIV) in red. Each domain contains six transmembrane segments (S1-S6). The segments S1-S4 form the voltage-sensing domain (VSD) and the segments S5-S6 with the segment P (P1 and P2) form the pore domain (PD). (B) Lateral view of the human Cav3.2 channel generated by homology modeling, the extracellular (EC), transmembrane (TM) and intracellular (IC) portion are indicated. (C) Extracellular view of the human Cav3.2 channel. (D) Extracellular view diagram of the spatial distribution of the transmembrane segments of the human Cav3.2 channel.
